# High natural gene expression variation in the reef-building coral *Acropora millepora*: potential for acclimative and adaptive plasticity

**DOI:** 10.1186/1471-2164-14-228

**Published:** 2013-04-08

**Authors:** Camila Granados-Cifuentes, Anthony J Bellantuono, Tyrone Ridgway, Ove Hoegh-Guldberg, Mauricio Rodriguez-Lanetty

**Affiliations:** 1 Department of Biology, University of Louisiana at Lafayette, Lafayette, LA, 70504, USA; 2 Department of Biological Sciences, Florida International University, Miami, FL, 33199, USA; 3 Oceanica Consulting Pty Ltd, PO Box 462, Wembley, WA, 6913, Australia; 4 The Oceans Institute, University of Western Australia, Crawley, WA, 6009, Australia; 5 ARC Centre of Excellence for Coral Reef Studies and Coral Genomics Group, School of Pharmacy and Molecular Sciences, James Cook University, Townsville, QLD, Australia; 6 Global Change Institute, The University of Queensland, St Lucia, QLD, Australia

**Keywords:** Coral reefs, Acropora millepora, Gene expression variation, Microarrays, Microsatellites, Intron, Epigenetics

## Abstract

**Background:**

Ecosystems worldwide are suffering the consequences of anthropogenic impact. The diverse ecosystem of coral reefs, for example, are globally threatened by increases in sea surface temperatures due to global warming. Studies to date have focused on determining genetic diversity, the sequence variability of genes in a species, as a proxy to estimate and predict the potential adaptive response of coral populations to environmental changes linked to climate changes. However, the examination of natural gene expression variation has received less attention. This variation has been implicated as an important factor in evolutionary processes, upon which natural selection can act.

**Results:**

We acclimatized coral nubbins from six colonies of the reef-building coral *Acropora millepora* to a common garden in Heron Island (Great Barrier Reef, GBR) for a period of four weeks to remove any site-specific environmental effects on the physiology of the coral nubbins. By using a cDNA microarray platform, we detected a high level of gene expression variation, with 17% (488) of the unigenes differentially expressed across coral nubbins of the six colonies (jsFDR-corrected, *p* < 0.01). Among the main categories of biological processes found differentially expressed were transport, translation, response to stimulus, oxidation-reduction processes, and apoptosis. We found that the transcriptional profiles did not correspond to the genotype of the colony characterized using either an intron of the carbonic anhydrase gene or microsatellite loci markers.

**Conclusion:**

Our results provide evidence of the high inter-colony variation in *A. millepora* at the transcriptomic level grown under a common garden and without a correspondence with genotypic identity. This finding brings to our attention the importance of taking into account natural variation between reef corals when assessing experimental gene expression differences. The high transcriptional variation detected in this study is interpreted and discussed within the context of adaptive potential and phenotypic plasticity of reef corals. Whether this variation will allow coral reefs to survive to current challenges remains unknown.

## Background

Symbiotic scleractinians provide the framework for coral reefs, one of the most diverse ecosystems in the world [1,2]. From a human perspective, coral reefs are extremely important as they provide billions of dollars to the communities in their vicinity [3,4]. From an evolutionary and ecological perspective, coral reefs house thousands of species and have been shaping the tropical waters of the oceans for 500,000 years [5-7]. As important and fascinating as they are, coral reefs are threatened by multiple stressors. Local factors such as pollution and overfishing are of particular concern [5]. Significantly, global factors also threaten coral reefs, including increased water temperature as a result of global warming, decreased oceanic pH resulting from an increase of atmospheric dissolved carbon dioxide in the oceans, and increased incidence of disease [5-9]. Studies at cellular, molecular, physiological, ecological, and evolutionary levels are imperative if we are to better understand whether coral reefs will survive the unprecedented increasing rates of CO_2_ and average seawater temperatures, as well as to provide solutions to management programs [10]. It is expected, then, that the persistence of coral reefs will depend upon the ability of reef corals to respond to these environmental stressors (e.g. [11]).

One important aspect in understanding how corals will respond to the aforementioned environmental stressors associated with global climate change is to examine the genetic diversity exhibited by these organisms. High genetic variation will mirror a diverse range of phenotypes, allowing populations to respond and adapt to changing environments and escape extinction [12]. However, recent advances in our understanding of gene expression variation in natural populations indicate that transcriptional variation might be also a possible mechanism to increase the repertoire of phenotypic variation upon which natural selection can act [13-16]. Gene expression variation has been tested within and between natural populations of model organisms including humans (e.g. [17,18]), fruit flies (e.g. [19]), the yeast *Saccharomyces cerevisiae* (e.g. [20]), killifish [14-16,21], Atlantic salmon [22,23], mice (e.g. [18,24]), and maize [18]. These studies have detected high and widespread gene expression variation among individuals within and between populations.

While several studies have examined the transcriptional response of a vast number of coral genes to different environmental stressors (e.g. [25-36]), there has been little focus on examining the natural gene expression variation of coral transcriptomes, with the exception of a few studies [37,38]. Interestingly, these studies did not detect large levels of transcriptional variation [37,38], although limitations in the experimental design of these studies preclude us to evaluate the gene expression variation occurring in nature that is not influenced by site-specific environmental effects. Therefore, it is still vital to further examine the level of natural variation in gene expression from coral populations as a way to understand its role within the adaptive potential and phenotypic plasticity of corals to global climate change. Moreover, it is necessary to examine the relationship between genotype and gene expression variation, as the response to stress of some genes might depend on the genetic composition [29] and this relationship has been under-explored in coral population genomics. Establishing this relationship will allow us to account for heritable mechanisms of potential natural gene expression variation in coral populations.

Our aim was to assess natural variation of coral gene expression and also, very importantly, to understand if there was a correspondence of colony genotype and transcriptional profiles of *Acropora millepora*. This reef-building coral species was implemented as our model system because of the large genomic, transcriptomic, and population genetic resources available: a draft genome sequence completed (released online prior to publication, http://coralbase.org/) and a great number of expressed sequence tags (EST) [39] useful for cDNA microarrays [40,41], and microsatellite loci (e.g. [42,43]). By bringing *A. millepora* coral nubbins to a common garden (i.e. under the same environmental conditions) in the reef lagoon of Heron Island (Great Barrier Reef, Australia), corals were allowed to acclimate to the same environment, removing environmental effects on the physiology of the coral nubbins. An intron of one of the carbonic anhydrase isoforms [Ridgway T, Hoegh-Guldberg O, Bongaerts P, Gresshoff PM, Riginos C: **Intron markers show evidence for cryptic divergence in sympatry in Acropora millepora on the southern Great Barrier Reef.** unpublished.] and microsatellite loci [43] were used as molecular markers to genotype the coral host. Transcriptional profiles of the coral nubbins were determined by microarray analysis. Given that the variation in gene expression did not correspond with either molecular intron or microsatellite genotypes, we account this considerable level of gene expression variation as a natural-occurring phenomenon in wild populations of reef corals.

## Results and discussion

### Genotypic identity in *A. millepora* as detected by a carbonic anhydrase-intron and microsatellite loci markers

We detected genotypic differences among 25 colonies tagged of *Acropora millepora* from the same reef flat on Heron Island (GBR) using the carbonic anhydrase 4–500 intron. We identified two different genotypes based on fingerprinting profiles (Additional file 1): 21 colonies as genotype 1 (displaying one single band of 550 bp) and four colonies as genotype 2 (showing two bands of 550 and 450 bp). To explore for natural gene expression variation between these two genotypes, we selected three colonies from each genotype for transcriptional profile comparison.

**Figure 1:**
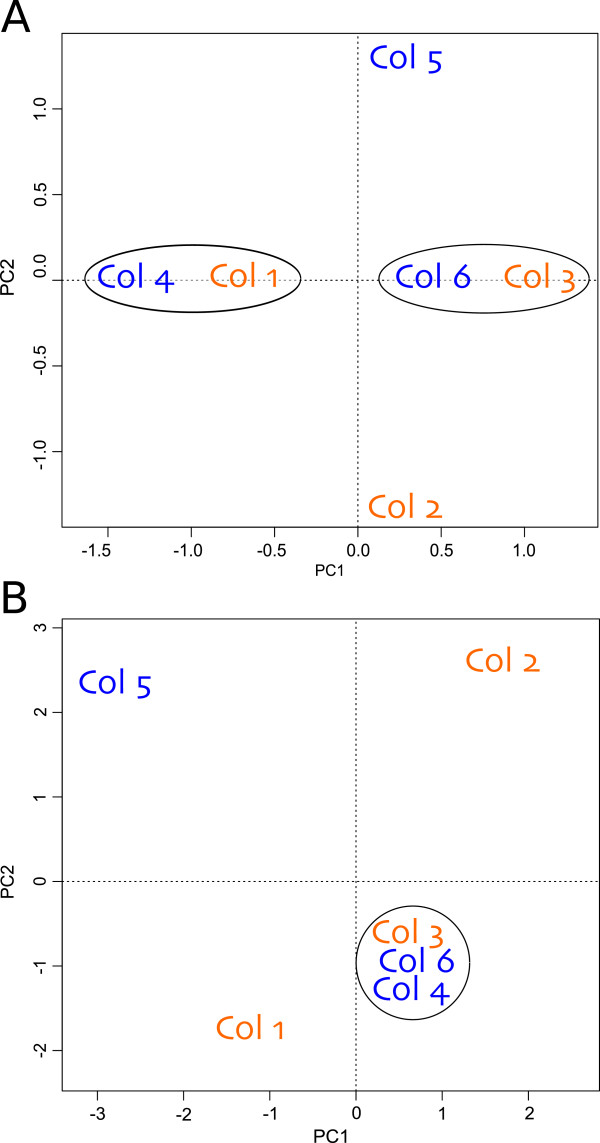
**Principal component analysis (PCA). A**) Ordination based on microsatellite loci genotypes (13 alleles). PC1 and PC2 explain 39% and 24% of the variation, respectively. **B**) Global pattern of gene expression on six colonies of *A. millepora*. PC1 and PC2 explain 44% and 24% of the variation, respectively. Orange = colonies genotyped as genotype 1 with the Intron 4–500. Blue = colonies genotyped as genotype 2 with the intron 4–500.

The colonies selected for transcriptional profile comparison were also genotyped using four microsatellite loci developed for *A. millepora* by van Oppen et al. [43] to further assess genotypic identity in the transplanted coral nubbins from the six colonies of *A. millepora* in the common garden. While we initially set out to screen a total of six microsatellite loci, two sets of microsatellite loci were not successfully amplified in all colonies. A total of 13 alleles were detected within the four screened microsatellite loci (Additional file 2), which is within the range of alleles (2–21 alleles per locus) identified in 947 colonies of *A. millepora* across the GBR using these genotypic markers [42]. Additional development of microsatellite markers using EST and whole-genome shotgun sequence (WGS) databases identified 40 polymorphic loci [44]. Similarly, the number of alleles ranged from two to 16 for EST microsatellites and from five to 18 for WGS microsatellites [44]. The alleles detected here are probably common in the southern GBR, as low levels of genetic flow have been described in this area [42]. Further examination of this data in a principal component analysis (PCA) allowed the detection of differentiation exhibited among colonies at the microsatellite level (Figure 1A). While we did not test for population structure (as this was beyond the scope of this study), the ordination of the colonies in the PCA according to microsatellites did not support the intron genotypes. Two clusters were resolved based on the axis of PC1, which explained almost 40% of the variation, but each cluster grouped colonies from the two intron genotypes. This highlights the importance of surveying genetic variation within a population of corals using different markers when carrying out molecular ecology studies (e.g. [45]).

### Transcriptomic variation among coral nubbins within a common garden

To determine the existence of natural gene expression variation and its correspondence with genotype given that some genes might respond based on the genetic background of the colony [29], transcriptional profiles of coral nubbins from six colonies (three from each of the two intron-genotypes) were determined using cDNA microarrays after bringing the coral nubbins to the same reef flat (common garden) for recovery and acclimation for four weeks. All colonies appeared healthy and coral nubbins were taken from the same tip position (see Methods). These procedures control for physiological differences between corals resulting from environmental sampling, allowing us to compare transcriptional statuses between colonies. A two-way ANOVA test in the reduced dataset found no differentially expressed genes (jsFDR-corrected; *p* > 0.05) in the coral colonies between the two intron genotypes. We also performed a mixed-ANOVA to detect variation in gene expression among all colonies. Unexpectedly, we detected a significant difference in gene expression, where 17% or 488 unique genes (1,021 features) from the cDNA microarray were differentially expressed between colonies (*p* < 0.01, jsFDR-corrected) (Additional file 3). While previous studies aimed at examining natural gene expression variation detected fewer differentially expressed genes (1.31% = 114 of 8686 genes, [38], 0.046% = 4 of 8686 genes [37]) than the study at hand, the experimental design of these studies prevented the examination of naturally occurring gene expression variation uninfluenced by environmental effects. In fact, this variation probably accounts for the variation (or lack thereof) in the level of gene expression of individual genes during heat stress [45,46]. As such, the large variation in natural gene expression detected here after controlling for environmental effects has not been previously reported and opens new questions and avenues of research in coral adaptive evolution and phenotypic plasticity, as further discussed below.

The proportion of genes differentially expressed in our study (17%) is comparable to other studies that measured gene expression in natural populations. For instance, 18% of the genes studied (161 genes) differed significantly between individuals of the same population of *Fundulus heteroclitus*[14]. In another study, 24% of the genes had significant expression levels among different strains of yeast [47]. Interestingly, we also found that the transcriptional difference among coral colonies was not attributed to the association with different types of *Symbiodinium*. Direct sequencing of the 28S nuclear rDNA from symbiotic dinoflagellates associated with the coral colonies showed that all colonies in the experiment harbored the same genetic type of *Symbiodinium* C3 (Acc. No. KC493130- KC493135).

To identify patterns in gene expression among colonies, a multidimensional ordination based on PCA was performed using the data of the differentially expressed genes (Figure 1B). Despite all coral nubbins acclimated to a common garden, the PCA ordination showed different transcriptional profiles among colonies, which highlights the importance of taking into account natural variation between colonies when assessing experimental gene expression differences. The first two axes explained 68% of the variation, where an important differentiation between the colonies was observed (Figure 1B). Colonies 3, 4, and 6 grouped together under both PC1 and PC2, and were separated by PC1 from colonies 1 and 5. Additionally, colonies 1, 3, 4, and 6 were separated from colonies 2 and 5 by PC2 (Figure 1B).

The PCA ordination also allowed the comparison of transcriptional statuses of the colonies with the two approaches implemented for colony genotyping. In the case of the intron genotype, the ordination of gene expression did not correspond to the two genotypes, corroborating the lack of statistical significance examined above between genotype and gene expression profiles. In the case of the microsatellites, there was not a clear correspondence between transcriptional state and colony genetic variation. However, some colonies showed partial congruence between genotype and gene expression. Both PCA ordinations (Figure 1A and 1B) showed colonies 3 and 6 in close graphical proximity to each other. The lack of correspondence between genotype and gene expression profiles is not unexpected, as previous studies have shown that the correspondence is not always straightforward. Environmental factors may have effects on the patterns of gene expression (reviewed by [48]) and this is seen in corals due to their branching pattern and colonial organization [37,49].

### General biological processes of differentially expressed genes

**Figure 2:**
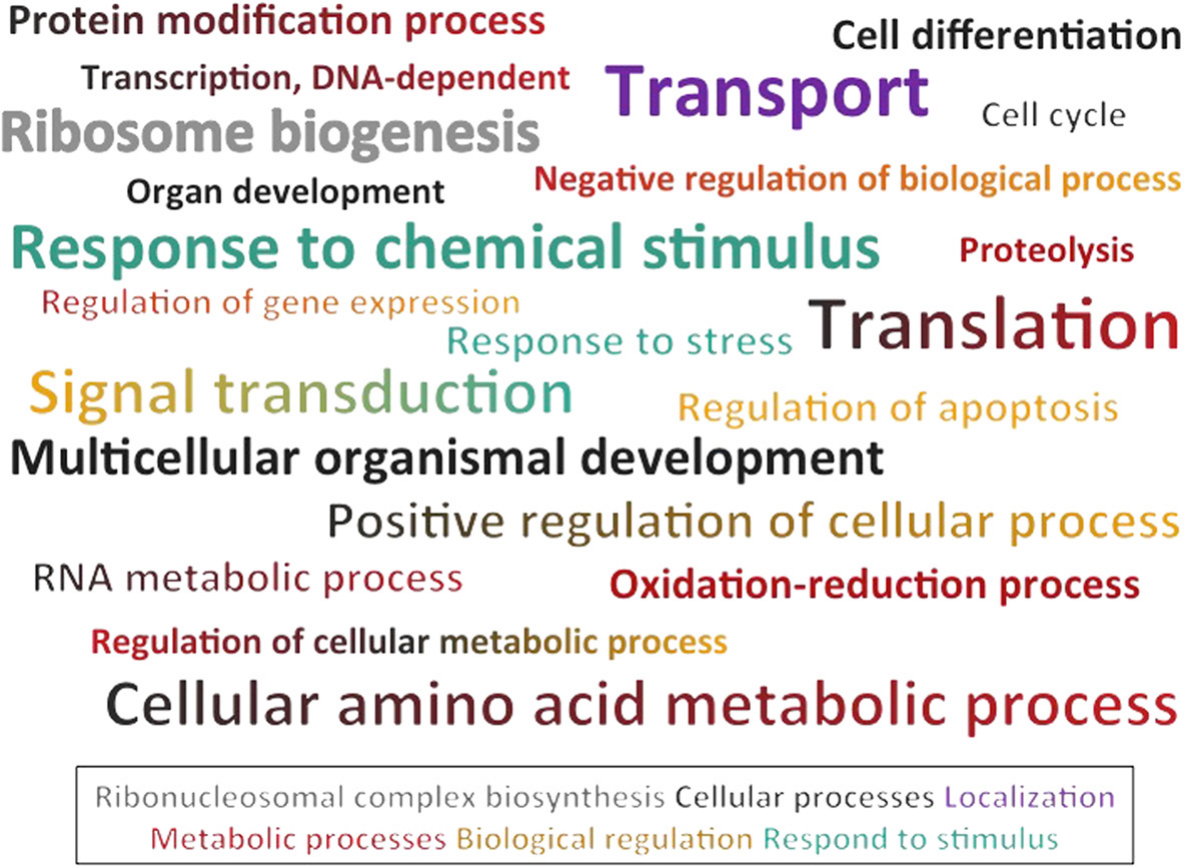
**Cloud-term representation of the biological process GO terms with node score >10 in the combined graph of Blast2GO.** The size of the font is proportional to the node score. GO terms with multiple colors indicated that more than one higher-level GO term was parent of the particular GO term.

Approximately 50% of the differentially expressed unigenes were successfully annotated in ~2,000 GO terms. The proportion of functional categories varied between colonies (Figure 2). The functions of transportation and translation have the highest annotation weight indicating that these GO terms had a large number of sequences and were closer to the term than other GO terms obtained. Previous studies on corals have shown various genes involved in transportation due to temperature effects (e.g. [25,26,32]), dark stress [50], the symbiotic relationship with *Symbiodinium*[51-53], metamorphosis and calcification [54], circadian clock regulation [55], and physiological plasticity [37,38]. Differential expression of translation has also been detected under different environmental stressors, including increase of temperature [26,30,32] and darkness [50], and associated to the life stage of corals [54] and the symbiosis with *Symbiodinium*[51]. We found that metabolic and cellular processes altered by the aforementioned factors are also naturally occurring in colonies of *A. millepora*.

### Oxidation-reduction processes

Coral cells are subject to elevated levels of oxygen radicals during sunlight hours, which are by-products of the photosynthetic reactions carried out by *Symbiodinium*[56]. These reactions cause the host to activate protective mechanisms for detoxification. Genes that exhibited variation in this category included *catalase*, *peroxiredoxin-mitochondrial-*like, *calcium/calmodulin-dependent kinase type II subunit delta*, and *ferritin*, as well as the lipid metabolic gene *sterol desaturase*. *Catalase* is a common enzyme in aerobic organisms utilized for the detoxification of hydrogen peroxide [57]. The gene coding for this enzyme has been shown as differentially expressed in corals under stress [26,30,32,45,56,58], correlated with *Symbiodinium* genotype [51], diel cycle [55], and metamorphosis and calcification [54]. *Peroxiredoxin* also reduces hydrogen peroxide [57], but the differential expression of this gene has not been detected in previous studies on corals. *Calcium/calmodulin-dependent protein kinase type II* is involved in the regulation of calcium homeostasis [57]. *Calmodulin* genes have been detected in the response of corals to the symbiotic state, i.e. whether the cnidarian host already established a symbiosis or not [52,53] as well as heat-stressed corals [25,26,30,32], calicoblast differentiation [54], and metamorphosis [59]. *Ferritin* is important for iron homeostasis and also has an oxidoreduction activity [57]. This iron-storage protein-coding gene has been differentially expressed in corals [26,30-32,60] and sea anemones [61] under thermal stress. Interestingly, EST libraries constructed from different life stages of *Acropora palmata* have identified *ferritin* as highly expressed [53]. Examination of these EST libraries and comparison with an *A. millepora* EST library revealed the presence of two types of ferritins [53]. To test for positive selection, Schwarz et al. [53] compared these EST libraries with a *Nematostella vectensis* database and found that the dN/dS ratio of ferritin type I is particularly high, indicating potential adaptive evolution.

### Genes involved in response to stress

Three GO terms have high node scores in the response-to-stimulus category: response to chemical stimulus, signal transduction, and response to stress. Some genes that were annotated with these GO terms are *heat shock protein 70* (*hsp70*), *catalase* (see above), *UV excision repair protein rad 23*, *ubiquitin* (see below), *ferritin* (see above), *peroxiredoxin* (see above), and *inhibitor nuclear factor kappa-beta* (*IκΒ*, see below). *Hsp70* helps stabilize preexisting proteins from aggregation and acts as a molecular chaperone, mediating new protein folding under normal and high temperature conditions [62]. *Hsp70* is shown to be up-regulated in heat-stressed corals [27,28,31,63-68] and is under a diel cycle [55]. However, some studies have not detected changes in gene expression of *hsp70* during thermal stress [32,33,46]. Given its well-documented molecular function in heat shock, the absence of *hsp70* amongst differentially expressed genes in some heat-stress studies could be due to the high level of variation that is naturally occurring as we demonstrated here, rather than due to a nonexistent response. Additionally, the timing at which the samples were collected or the use of the constitutive rather than the inducible gene could have potentially influenced this lack of detection in other studies.

Other interesting genes involved in the response to stimulus category are the genes involved in immune response. Cnidarians are thought to possess only an innate immune system, lacking an adaptive immune system as the one described in jawed vertebrates (for a review of immune phylogeny see [69]). One gene found differentially expressed among the colonies is the *inhibitor of the nuclear factor kappa-beta* (*IκΒ*). The detection of *IκΒ* suggests the negative regulation of the *nuclear factor kappa-beta* (*NF-κΒ*) pathway, involved in different biological processes including inflammation, immunity, and apoptosis [70]. It has been shown that *IκΒ* is under a diel cycle [55], is a potential candidate gene for regulation of the symbiosis cnidarian-*Symbiodinium*[71], and may be involved in thermal tolerance [33].

Another key gene is *ubiquitin*, possessing a critical role in protein turnover by labeling proteins for destruction [57]. In fact, proteolysis was a GO term with strong node support (Figure 2). Differential expression of *ubiquitin* has been observed by corals under stress [25-27,30,32,50,62,65] as well as during the establishment of the symbiosis between host and *Symbiodinium*[71].

The *myocyte-specific enhancer factor 2C* (*Mef2C*) is another differentially expressed immune gene associated with apoptosis. However, *Mef2C* is involved components of the adaptive immune system [72,73], which is not found in corals. Therefore, these genes probably evolved a different, as yet unknown function and demand further exploration.

### Differential expression of apoptotic genes

Apoptosis has received attention in coral physiology, given that it is one possible mechanism by which corals undergo bleaching (reviewed by [74]). Some genes annotated with this GO term include *ras-like GTP-binding protein rho1* and *CCAAT/Enhancer binding protein* (*C/EBP*) gamma, which have been previously detected in stressed corals and anemones, and in response to symbiosis [26,30,50,53,54,61]. Interestingly, the C/*EBP* was found to be stable in a study on coral thermo-tolerance [33] and expressed throughout different life stages [53]. We detected differential expression of *glyceraldehyde 3-phosphate dehydrogenase* (*GAPDH*). However, *GAPDH* was not differentially expressed during natural bleaching [45] and is proposed as a potential housekeeping gene [75].

A *B-cell lymphoma protein-2 like-2* (*Bcl*-2) gene, which promotes cell survival by suppressing the activity of *Bcl-2-associated X* (*Bax*) [76], was differentially expressed. Additionally, *Bax* was also inhibited by another differentially expressed gene: a probable *bax inhibitor 1*. This variation suggests that corals repress apoptosis under normal physiological conditions, in the absence of what is typically deemed heat stress. For example, colonies of *A. millepora* undergoing heat stress showed evidence of induction of apoptosis during thermal stress with a delayed up-regulation in *Bcl-2* (anti-apoptosis) of surviving cells as a protective mechanism [77]. Stressed colonies of a congener species, *A. palmata*, showed up-regulation of an anti-apoptosis Bcl-2 family member [25], supporting the hypothesis that anti-apoptosis members protect surviving cells. This anti-apoptotic activity has also been detected during coral metamorphosis [59].

Finally, the *MAPK MAK MRK overlapping kinase* or *MOK* was another differentially expressed gene within the apoptosis GO term. *MOK* is a member of the mitogen-activated protein kinases (MAPKs) [57]. MAPKs are involved in signal transduction pathways integrating different biological processes, such as immune response to pathogen infection, exocytosis, and redox signaling (e.g. [78,79]). Genes of this class have important function in the symbiosis of reef corals and *Symbiodinium*. For example, it has been hypothesized that regulation of MAPK-pathway members *sphingosine* and sphingosine-1-phosphate (*S1P*) allows the host cell containing the algae to survive and proliferate [52]. In fact, EST libraries and microarray data for *A. palmata* and *M. faveolata* confirm the importance of MAPK signaling in host-*Symbiodinium* symbiosis [53,71]. MAPKs may also be involved during coral bleaching in the process of *Symbiodinium* exocytosis [80] and osmoregulation [81]. Interestingly, the expression of an MAPK member, tribble, also exhibited high inter-colony variation during a natural bleaching event [45].

### Adaptive potential and phenotypic plasticity in corals

Overall, there was no correspondence between transcriptional expression profiles with either intron or microsatellite genotypes in coral colonies grown under a common garden (Figure 1). However, the high level of gene expression variation revealed might be a natural-occurring phenomenon in wild populations of reef corals. In light of these results, two significant questions arise. Firstly, what are the sources/mechanisms driving differences in the gene expression detected in colonies of *Acropora millepora* acclimatized in a common garden? Secondly, what is the importance of this natural gene expression variation within an ecological and evolutionary context?

Within the genome, polymorphic sites can alter transcriptional rates [82-85], contributing to additional variation at the mRNA level. Gene expression can also be altered through epigenetic modifications influenced by the environment (reviewed by [86-90]). In the case of corals, the role of epigenetics is currently unknown, but may explain some instances of acquired long-term stress tolerance (e.g. [91,92]). Moreover, it could provide a framework to explain the role of natural gene expression variation in corals within an ecological and evolutionary context.

Most of the differentially expressed genes identified in this common garden experiment have been implicated in coral stress (e.g.[25,26,28-33,45,50,60,63,67]) and possible resilience response [33] to environmental factors, some of which are linked to global climate changes, including ocean warming. A number of these environmental drivers might be able to trigger epigenetic changes in corals generating a mosaic of transcriptional diversity within populations. This transcriptional diversity could be an important source for evolution, probably more than protein isoforms as it has been previously suggested [14-16,20-23,93]. This raises the urgent need to explore the existence of epigenetic changes in coral and its role in the physiological and adaptive response to environmental changes.

It is well-known that coral reefs face a challenging future with conditions predicted to change, including an increase in temperature, a decrease in pH, and outbreaks of disease [5,7,9,94,95]. Although the response to some of these conditions might be similar across colonies (e.g.[36]), variation occurring at the transcriptomic level is vital for stress response (reviewed by [96,97]). Numerous studies have demonstrated that the so-called ‘core stress response’ could explain why cells can resist different stresses if they were previously treated with low levels of one stress factor [96,97]. Therefore, the differential gene expression generated during a specific stress is not directed towards that particular challenge, but rather form part of the generalized core stress response [96]. In fact, a recent study from our research group has showed that corals pre-exposed to sub-bleaching temperatures are able to resist bleaching by changing the magnitude of the expression levels of differentially expressed genes [33]. Fascinatingly, it has been determined that genes with a TATA box in their promoters have an increased number of binding sites for transcription factors, which increases their sensitivity when in need of being transcribed [85]. Here, we detected differentially expressed genes like *hsp70*, *catalase*, *ubiquitin*, and *ferritin*, probably genes of the core stress response of corals. An interesting avenue of research is to determine if stress-related genes containing TATA boxes in corals also exhibit rapid regulatory evolution.

## Conclusions

In this study, we were able to genotype colonies of *Acropora millepora* from the reef flat surrounding Heron Island (GBR) by a high-resolution marker, microsatellites, and an additional molecular marker, intron 4–500 of a carbonic anhydrase isoform. The latter identified two different genotypes. We further explored the transcriptomic variation of six colonies by acclimatizing coral nubbins to a common garden in the same reef flat. Although no correspondence between transcriptional profiles and colony genotype was found (Figure 1), we revealed substantial natural gene expression variation occurring in these acclimatized coral nubbins. Some of the differentially expressed biological processes include transport and translation (Figure 2); these processes have previously been identified in other studies of corals examining the transcriptomic variability to various experimental factors, as well as natural variation (e.g.[25,26,32,37,38,50,55]). Genes in the category of oxidation-reduction process were also differentially expressed, most likely as a consequence of the photosynthetic activity of the dinoflagellate symbiont [56]. Genes involved in response to stimulus were also differentially expressed among colonies. This category contained several stress genes, including immune response genes. The considerable expression variation highlights the normal individual variation of coral colonies. Therefore, studies exploring gene expression either in response to stress or natural variation must consider natural variation occurring between individuals.

Importantly, natural gene expression variation could be the raw material upon which natural selection can act for evolution. Furthermore, this variation at the transcriptomic level combined with epigenomic modifications may be a source of phenotypic plasticity, which could potentially allow reef corals to respond to changing environments. Whether these genetic and epigenetic responses of corals and its symbionts will allow coral reefs to cope with the rapid pace of global change remains unknown.

## Methods

### Sample collection

Twenty-five colonies of *Acropora millepora* located on the reef flat of Heron Island, GBR, Australia (23°33'S, 151°54'E) were tagged in June 2007. This reef flat was within an area of 60,000 m^2^ and depth ranging from 0.5 to 2 m. There were no physical measurements. Coral fragments from these colonies were sampled for host and symbiotic dinoflagellate DNA genotyping, as explained below. After host genotyping, branches from six colonies belonging to two different genotypes (three colonies per genotype), as detected by the same marker Intron 4–500 (see below), were fixed in marine epoxy in 15 ml cut-off centrifuge tubes and brought to a common garden. These six colonies appeared healthy, with no visible signs of disease. The coral nubbins were left to recover and acclimatize in this common garden of 2 m^2^ in a depth that fluctuated due to tidal changes between 0.8 to 2 m for a period of four weeks. The purpose of this was the removal of any site-specific environmental effects on the physiology of the coral nubbins, as well as to allow recovery from handling stress. After this period, three coral fragments 3 cm in length collected from the top of the colony of each of the coral nubbins were collected and snap-frozen in liquid nitrogen for later DNA and RNA isolation. Collections from the same position in all the colonies would reduce potential variability due to branch variation, and therefore allow straight comparisons between colonies.

### DNA extraction and host genotyping

DNA extractions on tissue from the 25-tagged colonies were carried out using a DNeasy Kit (QIAGEN) following the manufacture instructions. DNA concentrations were measured using a NanoDrop ND-1000 UV–vis Spectrophotometer (Nano-Drop Technologies) The intron marker 4–500 of one of the three isoforms of carbonic anhydrase [Ridgway T, Hoegh-Guldberg O, Bongaerts P, Gresshoff PM, Riginos C: **Intron markers show evidence for cryptic divergence in sympatry in Acropora millepora on the southern Great Barrier Reef.** unpublished.] was initially used for genotyping the colonies. Given that hybridization and gene introgression are a possibility in corals, the sequencing of DNA may still offer adequate power to distinguish intra-population structure because the underlying mutational models (e.g. neutrality test) are well understood and homoplasy among alleles/haplotypes should be low. The 4–500 CA intron region was amplified using the forward primer ‘4-5 F’ (5’- TCC CCG GAA CGT TCA CAA CTG CTC-3’) and ‘4-5R’ (5’-CAA CAT CAA GTA TGG GGG CAT T-3’) [Ridgway T, Hoegh-Guldberg O, Bongaerts P, Gresshoff PM, Riginos C: **Intron markers show evidence for cryptic divergence in sympatry in Acropora millepora on the southern Great Barrier Reef.** unpublished.]. Coral-host specificity of the intron was confirmed via PCR amplification of *Symbiodinium*-free-DNA isolated from *A. millepora* sperm, combined with a lack of amplification from DNA obtained from *Symbiodinium* cultures [Ridgway T, Hoegh-Guldberg O, Bongaerts P, Gresshoff PM, Riginos C: **Intron markers show evidence for cryptic divergence in sympatry in *Acropora millepora *on the southern Great Barrier Reef.** unpublished.]. PCR amplifications from the tagged colonies were performed with the following conditions: 1.0 μl of DNA template at a concentration between 12.5 and 31.3 ng/μl, 12.5 μl of GoTaq Green Master Mix 1X (2X Green GoTaq reaction buffer, 400 μM each dNTP, 3.0 mM MgCl_2_, GoTaq DNA polymerase, Promega), 0.25 μM of each primer, and added Milli-Q water for a final volume of 25 μl per reaction. The PCR program consisted of an initial denaturing step at 94°C for 3 min, 35 cycles at 94°C for 20s, 54.2°C for 20s, and 72°C for 1 min, and a final extension at 72°C for 10 min. PCR products were run on 1% TBE agarose gels to confirm amplification and size of PCR product. Samples that exhibited one band were sent to the DNA Analysis Facility at Yale University for purification and sequencing in both, the forward and reverse directions. Sequences were verified and edited in CodonCode Aligner (CodonCode Corp.). Samples that exhibited two bands were cloned into pGEM-T Easy Vector System I (Promega), and plasmids were purified using the UltraCleanTM Plasmid Prep Kit (MO BIO Laboratories). Plasmid inserts were sequenced bidirectionally at the Australian Genome Research Facility (ABI BigDye Terminator chemistry v3.1). Chromatograms from these sequences were visually checked using the program Seqman (Lasergene), and aligned using MacClade 4 [98]. BLAST was used to search NBCI’s Genbank to confirm the carbonic anhydrase isoform. Differences in nucleotides were viewed in CLC Sequence Viewer (CLC bio). Final edited sequences were deposited in Genbank (Acc. No. KC493136-KC493137).

Further genotyping, was subsequently performed using microsatellite loci developed for *A. millepora*[43]. A total of six microsatellite loci were tested (Amil2_002, Amil2_006, Amil2_007, Amil2_010, Amil2_012, Amil2_022). Forward primers have a 5’-universal M13 tail for labeling using the protocol of [99]. DNA was amplified according to [43]. Briefly, PCR reactions contained 3 μl of DNA template at a concentration of 10 ng/μl, 1X PCR buffer (Promega), 1.5 mΜ MgCl_2_ (Promega), 200 μΜ dNTPs (Invitrogen), 0.04 pmol of the 5’ M13-tailed forward primer, 0.16 pmol reverse primer and FAM-labeled universal M13 primer, 0.25 U of HotStart *Taq* polymerase (Promega), and brought up to a final reaction volume of 20 μl with nuclease-free water. PCR conditions consisted of an initial denaturing step at 94°C for 5 min, 30 cycles at 94°C for 30 s, 56°C for 45 s, 72°C for 45 s, 8 cycles at 94°C for 40 s, 53°C for 45 s, 72°C for 45 s, and a final extension cycle at 72°C for 15 min. Microsatellites Amil2_007 and Amil2_012 did not amplify and were not included in further analysis. Samples were sent to the DNA Analysis Facility at Yale University for purification and sequencing, with automated sequencing performed with on a 3730xl 96-Capillary Genetic Analyzer with the Liz-500 size standard. A principal component analysis (PCA) was run using the software R/VEGAN v.1.17-3 [100,101] in order to obtain a visual ordination of the microsatellite haplotypes.

### *Symbiodinium* genotyping

We examined the composition of *Symbiodinium* associated with the studied corals grown under the same common garden, as it has been shown in previous studies that coral host transcriptional response varies in function of the type of symbiont associated [51]. For this, the variable domains D1 and D2 of the 28S nuclear rDNA were amplified with the following primers: TohaF 5’-CCT CAG TAA TGG CGA ATG AAC A-3’ and TohaR 5’-CCT TGG TCC GTG TTT CAA GA-3’ [102,103]. DNA extractions contained between 11–76 ng/μl. PCR amplifications were carried out under the following conditions: one cycle at 94° for 5 min, 30 cycles at 94°C for 1 min, 65°C for 2 min, and 72°C for 3 min, and a final extension for 10 min at 72°C. Amplifications of the expected size (~800 bp) were purified with QIAquick PCR Purification Kit (Qiagen) according to manufacturer’s protocol and directly sequenced Australian Genome Research Facility (ABI BigDye Terminator chemistry v3.1). BLAST searches in NCBI’s Genbank were carried out to determine the identity of the sequences. Sequences were a direct match (100% similarity) with *Symbiodinium* C3.

### Hybridization of microarrays

To measure gene expression variation from the acclimatized colonies in the common garden, a cDNA microarray platform comprised of 8,386 unigenes developed in collaboration by two coral genomics research groups from the Australian National University and James Cook University [40] was used. Details of the fabrication of microarrays are explained by Grasso et al. [41]. This microarray platform has been successfully implemented to address multiple biological questions over the past five years [31,33,37,38,41,55]. We applied a multiple dye-swap microarray design (Additional file 4) for the two groups of coral colonies (N = 3 per group) defined based on the two genotypes resolved with the use of intron 4–500. Variation between the colonies was also examined by carrying out a loop design (Additional file 4). Three nubbins per colony were included in each loop within each intron genotype.

### RNA extraction, cDNA synthesis and microarray hybridizations

For probe construction, total RNA from the acclimatized colonies was extracted using Trizol (Invitrogen) and an RNeasy Mini Kit (QIAGEN), following the manufacture’s instructions. The integrity and quality of RNA was assessed using a Bioanalyzer (Agilent Technology). The concentration of RNA was also measured using a NanoDrop ND-1000 UV–vis Spectrophotometer (Nano-Drop Technologies). cDNA probe synthesis was performed from samples showing intact RNA (2.5 μg total RNA) using Superscript II Reverse Transcriptase (Invitrogen) and the 3DNA Array 350 kit (Genisphere) according to the manufacturer’s guidelines. The arrays were initially prehybridized and then hybridization was performed in two steps: 1) cDNA hybridization, where cDNA is first hybridized to the spotted microarray, at 47°C for 14 h, and 2) 3DNA hybridization, where dyes hybridize to the reverse transcribed samples labeled with Cy3 and Cy5, at 50°C for 4 h. Prehybridization and hybridization were done following manufacture’s protocol (Genisphere). Washes were performed between the prehybridization and the two steps of hybridization, as well as before signal detection. Washes consisted of 15 min in 2X SSC, 0.2% SDS at 42°C, 15 min in 2X SSC at room temperature, and 15 min in 0.2X SSC at room temperature. Slides were immediately transferred to a dry 50 mL centrifuge tube and centrifuged for 2 min at 800 rpm to dry the slide. After the last wash, microarray slides were submerged in dye saver (Genisphere) for 5 s and dried via centrifugation as described above. Slides were scanned using an Axon GenePix 4000 scanner (Molecular Devices) and the software GenePix Pro (Molecular Devices) was used to extract the intensity values. The data discussed in this publication have been deposited in NCBI’s Gene Expression Omnibus [104] and are accessible through GEO Series accession number GSE42684 (http://www.ncbi.nlm.nih.gov/geo/query/acc.cgi?acc=GSE42684).

### Analysis of microarray data

Data was initially filtered from 18,142 to 6000 data points to reduce background noise from the microarray data and the effect of spots with intensity signals at the lower limits of detection. Subsequent analyses were performed on this reduced dataset. A logarithmic transformation of raw intensity data to ratios was carried out to account for sources of variation in the data that are not due to differential expression of genes. Ratio-intensity (RI) plots were constructed for each set of array data to explore whether or not intensity dependence of log ratios (which appears as curvature) was present. Because curvatures were detected, an rLowess curve fitting transformation [105] was applied to all arrays. In order to detect differentially expressed genes, a two-way ANOVA and a mixed ANOVA were performed on the transformed data using the software R/MAANOVA v2.10.1 [101,106] between genotypes and among all colonies, respectively. Each dye and array were considered in the model as fixed and random factors, respectively. P-values from the F-test (500 permutations) were adjusted for type I error using John Story’s false discovery rate (jsFDR) [107]. Only genes with adjusted P-values less than 0.01 were considered differentially expressed. To explore the patterns of gene expression in a multi-dimensional ordination after redundant genes were removed, a PCA was conducted also using the software R/VEGAN v.1.17-3 [100,101].

The suite Blast2GO [108] was used to determine the functional, biological, and cellular components of the genes detected as differentially expressed. We used a non-redundant dataset. Blast2GO uses the engine BLAST to search sequences in Genbank (NCBI), map, and annotate genes to gene ontology (GO) categories. The annotation in Blast2GO is based on similarity searches using a statistical framework. Additional annotation by InterProScan and Annex augmentation were also incorporated, as they improve annotation by increasing the number of significant figures [109]. In order to determine enriched terms (here defined as those GO terms that have a high node score), a combined graph was obtained for GO terms that belonged to the “Biological Process” category with a node score >10 as annotation weight. This node score considers the number of sequences converging at a particular GO term, but penalizes the distance of the sequence to that GO term [108]. From this graph, a cloud-term figure was created (e.g. [110]). Higher-level GO terms were obtained from the *Saccharomyces* Genome Database [111].

## Competing interests

The authors declare that they have no competing interests.

## Authors’ contributions

CGC carried out the molecular genetic study, performed the statistical analyses, gene database blasts, and drafted the manuscript. AJB was involved in effecting the statistical analyses. TR participated in the field and part of sampling processing. OHG participated in the conceptualization and design of the study. MRL conceived the study, participated in the design and coordination of the study, and performed part of the molecular genetic study. All the authors read and approved the final manuscript.

## Supplementary Material

Additional file 1 **Fingerprinting profile of 25 colonies of *A. millepora *from Heron Island GBR) using intron 4-500 (agarose, TBE 1%).** Colonies selected as genotype 1 and genotype 2 for transcriptomic analyses are depicted in orange and blue, respectively. L = 100-1000 bp size ladder, NTC= no-template control. Click here for file

Additional file 2 Matrix of microsatellite data showing the presence/absence of the alleles of each microsatellite for all the coral colonies. Click here for file

Additional file 3 **Known unigenes showing significant differences between coral colonies of *A. millepora*.** Identification was based on BLAST hits (E values < 10^-5^). The molecular function of the hit is shown, as obtained from Blast2GO. Significance was determined at *P* < 0.01, js-FDR corrected). “-“ indicates that there was no information regarding the molecular function of that particular BLAST hit. Click here for file

Additional file 4 **Design of the microarray experiments.** Dye swap was performed between colonies of the two intron 4-500 genotypes (shaded). Loop design was performed three times with different nubbins of each colony within each genotype. A total of 22 microarrays were used, as a replicate of one of the colonies of genotype 2 did not yield enough RNA. Colonies 1, 2, and 3 were genotyped as genotype 1 with intron 4-500. Colonies 4, 5, and 6 were genotyped as genotype 2 with intron 4-500. Click here for file
