# Supplementary figures and images for: High natural gene expression variation in the reef-building coral Acropora millepora: potential for acclimative and adaptive plasticity
Source: BMC Genomics. 2013 Apr 8;14:228. doi: 10.1186/1471-2164-14-228 (PMC3630057; doi:10.1186/1471-2164-14-228)

L NTC 1 2 3 4 5 6 7 8 9 10 11 12 13 14 15 16 17 18

L 19 20 21 22 23 24 25

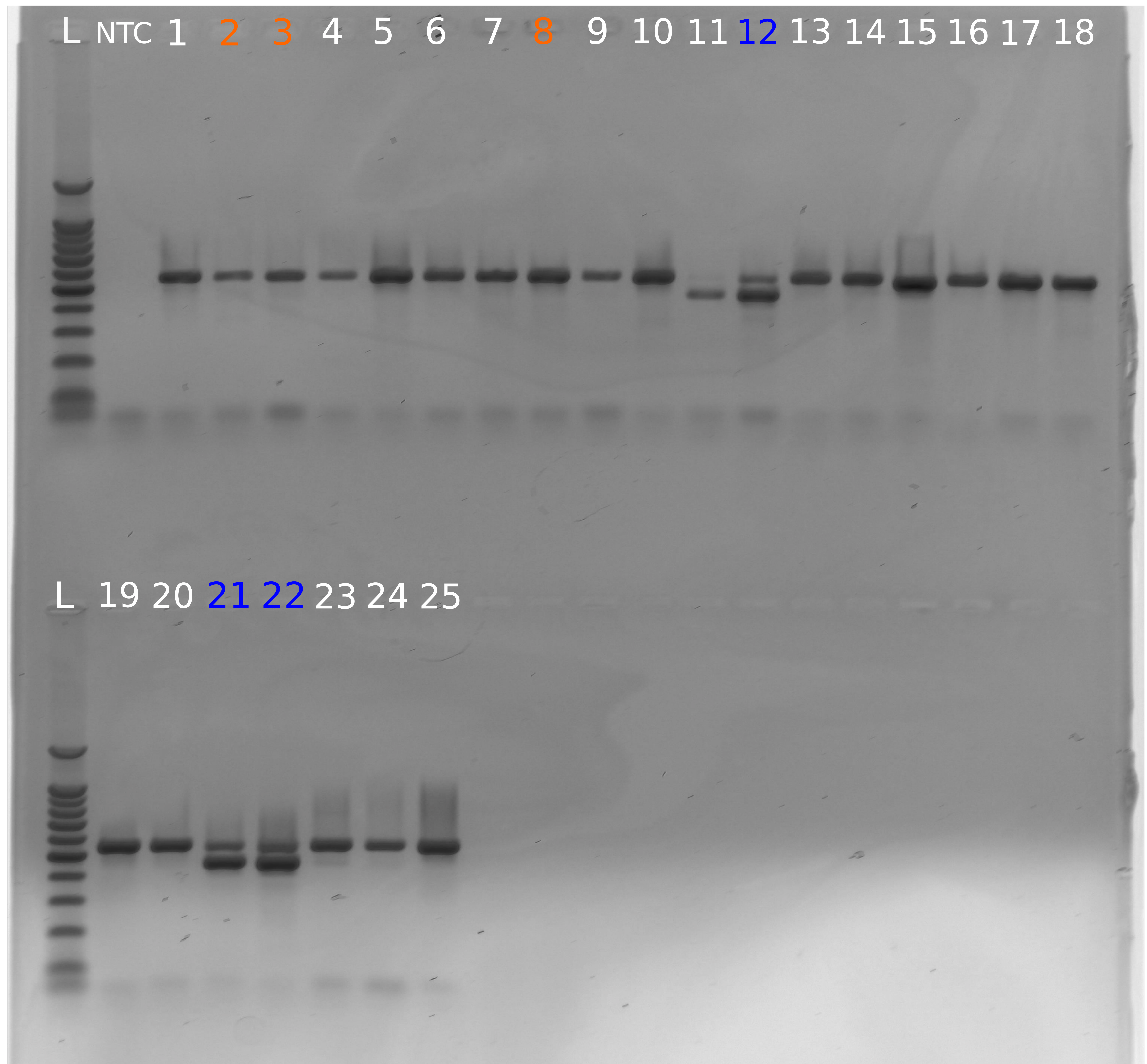

Supplement: Additional file 1 — Fingerprinting profile of 25 colonies of A. millepora from Heron Island GBR) using intron 4-500 (agarose, TBE 1%). Colonies selected as genotype 1 and genotype 2 for transcriptomic analyses are depicted in orange and blue, respectively. L = 100-1000 bp size ladder, NTC= no-template control. [file 1471-2164-14-228-S1.pdf]
