# Supplementary material for: High natural gene expression variation in the reef-building coral Acropora millepora: potential for acclimative and adaptive plasticity
Source: BMC Genomics. 2013 Apr 8;14:228. doi: 10.1186/1471-2164-14-228 (PMC3630057; doi:10.1186/1471-2164-14-228)
Supplement: Additional file 2 — Matrix of microsatellite data showing the presence/absence of the alleles of each microsatellite for all the coral colonies. [file 1471-2164-14-228-S2.docx]

**Additional file 2 – Matrix of microsatellite data showing the presence/absence of the alleles of each microsatellite for all the coral colonies**

| Microsatellite | M10 | M10 | M10 | M10 | M10 | M10 | M22 | M22 | M22 | M6 | M6 | M2 | M2 |
| --- | --- | --- | --- | --- | --- | --- | --- | --- | --- | --- | --- | --- | --- |
| colony/allele | a1 | a2 | a3 | a4 | a5 | a6 | a7 | a8 | a9 | a10 | a11 | a12 | a13 |
| col 1 | 0 | 0 | 1 | 1 | 0 | 0 | 1 | 0 | 0 | 1 | 0 | 1 | 0 |
| col 2 | 1 | 0 | 0 | 0 | 0 | 1 | 1 | 0 | 1 | 1 | 0 | 1 | 0 |
| col 3 | 1 | 1 | 0 | 0 | 0 | 0 | 1 | 0 | 1 | 1 | 1 | 1 | 1 |
| col 4 | 0 | 1 | 0 | 0 | 1 | 0 | 1 | 0 | 0 | 1 | 1 | 1 | 0 |
| col 5 | 0 | 0 | 0 | 1 | 1 | 0 | 0 | 1 | 1 | 1 | 0 | 1 | 0 |
| col 6 | 1 | 1 | 0 | 0 | 0 | 0 | 1 | 0 | 0 | 1 | 0 | 1 | 0 |
