# Supplementary material for: High natural gene expression variation in the reef-building coral Acropora millepora: potential for acclimative and adaptive plasticity
Source: BMC Genomics. 2013 Apr 8;14:228. doi: 10.1186/1471-2164-14-228 (PMC3630057; doi:10.1186/1471-2164-14-228)
Supplement: Additional file 3 — Known unigenes showing significant differences between coral colonies of A. millepora. Identification was based on BLAST hits (E values < 10-5). The molecular function of the hit is shown, as obtained from Blast2GO. Significance was determined at P < 0.01, js-FDR corrected). “-“ indicates that there was no information regarding the molecular function of that particular BLAST hit. [file 1471-2164-14-228-S3.docx]

**Additional file 3 – Known unigenes showing significant differences between coral colonies of *A. millepora*.**

Identification was based on BLAST hits (E values < 10^-5^). The molecular function of the hit is shown, as obtained from Blast2GO. Significance was determined at *P* < 0.01, js-FDR corrected). “-“ indicates that there was no information regarding the molecular function of that particular BLAST hit.

| **Sequence Name** | **BLAST Hit** | **GO Molecular Function** | ***P* - value** |
| --- | --- | --- | --- |
| C_D003-C7 | cathepsin d | Aspartic-type endopeptidase activity | 0.000150061 |
| C_D003-D2 | cytoplasmic actin | ATP binding | 0.007657128 |
| C_D003-E12 | protein | GTPase activity; GTP binding | 0.000576279 |
| C_D004-A11 | protein | Binding | 0.000721559 |
| C_D004-E6 | histone -like | DNA binding | 0.0000515 |
| C_D004-G12 | ras-like GTP-binding protein rho1 | Signal transducer activity; GTP binding; myosin binding; GTPase activity | 0.000368122 |
| C_D006-B2 | profilin | Actin binding | 0.008525026 |
| C_D006-E5 | calcium-transporting ATPase type 2c member 2-like | Calcium-transporting ATPase activity; manganese-transporting ATPase activity | 0.005958522 |
| C_D008-B12 | glucose sorbosone dehydrogenase | Binding | 0.000133424 |
| C_D008-C4 | splicing factor 3a subunit 2 | Nucleic acid binding; zinc ion binding | 0.000579574 |
| C_D009-C6 | gelsolin | Calcium ion binding; actin binding | 0.007263225 |
| C_D009-C9 | transcription factor ap-1 | Sequence-specific DNA binding transcription factor activity; sequence-specific DNA binding; protein dimerization activity | 0.000174878 |
| C_D009-D7 | cathepsin l | Cysteine-type endopeptidase activity | 0.0000515 |
| C_D009-F2 | actin-related protein 2 3 complex subunit 1a | Actin binding | 0.001178451 |
| C_D010-A6_41 | transcriptional regulator erg | Signal transducer activity; protein binding; delayed rectifier potassium channel activity; DNA binding | 0.000133424 |
| C_D010-C10_75 | nuclear factor nf-kappa-b p105 subunit | NF-kappaB binding | 0.001066522 |
| C_D010-C5_35 | probable bax inhibitor 1 | - | 0.000530389 |
| C_D010-H10_80 | receptor expression-enhancing protein 5-like | Protein binding | 0.006862988 |
| C_D011-A6_41 | protein | Protease binding; protein heterodimerization activity; protein homodimerization activity; BH3 domain binding; channel activity; sequence-specific DNA binding | 0.001038895 |
| C_D011-E7_53 | early growth response 1 | Nucleic acid binding; zinc ion binding | 0.000188911 |
| C_D013-C10_75 | a chain carbonic anhydrase ii inhibitor | Carbonate dehydratase activity; zinc ion binding | 0.0000131 |
| C_D016-D12_92 | glycine-rich rna binding protein | Binding | 0.001222495 |
| C_D017-G10_79 | cdgsh iron sulfur domain-containing protein 1 | 2 iron, 2 sulfur cluster binding | 0.0000725 |
| C_D018-C8_59 | protein isoform a | Protein binding | 0.008057598 |
| C_D018-F4_30 | ribosomal protein sa | Structural constituent of ribosome; laminin receptor activity; ribosome binding; laminin binding | 0.000371269 |
| C_D020-B5_34 | mucosa-associated lymphoid tissue lymphoma translocation protein 1-like | Peptidase activity | 0.000716396 |
| C_D020-F6_46 | adp-ribosylation factor 1 | Receptor signaling protein activity; protein binding; GTP binding; GTPase activity | 0.001325587 |
| C_D021-D7_52 | thioredoxin-1 | Catalytic activity | 0.002121557 |
| C_D021-H3_24 | lysosome-associated membrane glycoprotein 1-like | - | 0.002396199 |
| C_D023-G10_79 | protein | Binding | 0.0000131 |
| C_D024-G1_7 | carbonic anhydrase | - | 0.003500417 |
| C_D026-G5_39 | solute carrier family facilitated glucose transporter member 3-like | Sugar transmembrane transporter activity | 0.00319536 |
| C_D027-D2_12 | actin-related protein 3 | Actin binding; ATP binding | 0.000764887 |
| C_D028-B5_34 | ferritin | Transition metal ion binding | 0.003100633 |
| C_D028-E7_53 | eukaryotic translation initiation factor 2 alpha kinase | Transferase activity | 0.007228698 |
| C_D028-F6_46 | selenium binding protein 1 | Selenium binding; protein binding | 0.002902867 |
| C_D030-A5_33 | is5 transposase and trans-activator | Transposase activity; DNA binding | 0.000626548 |
| C_D032-E7_53 | nuclear pore complex protein nup155 | Structural constituent of nuclear pore; protein binding; transporter activity | 0.009574988 |
| C_D033-H11_88 | ets-1 p27 | Sequence-specific DNA binding; sequence-specific DNA binding transcription factor activity; glucocorticoid receptor binding; transcription factor binding | 0.0000288 |
| C_D034-F10_78 | protein | - | 0.003497137 |
| C_D035-D4_28 | proteasome subunit alpha type-6-like | Purine ribonucleoside triphosphate binding; threonine-type endopeptidase activity; NF-kappaB binding; RNA binding | 0.001645376 |
| C_D038-G5_39 | tumor rejection antigen 1 | Virion binding; calcium ion binding; low-density lipoprotein particle receptor binding; unfolded protein binding; protein phosphatase binding; RNA binding; ATP binding | 0.000471441 |
| C_D039-A10_73 | adaptor-related protein complex mu 1 isoform cra_a | Protein binding; transporter activity; lipid binding | 0.002665296 |
| C_D039-F6_46 | periostin isoform 2 | - | 0.002207931 |
| C_D040-B2_10 | rbm3 protein | Nucleic acid binding; ribosomal large subunit binding; protein binding | 0.008259768 |
| C_D040-D8_60 | protein | Catalytic activity | 0.0000623 |
| C_D041-F4_30 | cub domain protein | Binding; metallopeptidase activity | 0.008259768 |
| C_D045-H2_16 | proactivator polypeptide precursor | - | 0.004393693 |
| C_D046-C9_67 | aaa ATPase containing von willebrand factor type a | - | 0 |
| C_D047-B5_34 | thioredoxin-1 | Catalytic activity | 0.0000131 |
| C_D048-A5_33 | lysine-specific demethylase 6a-like | Metal ion binding; oxidoreductase activity, acting on single donors with incorporation of molecular oxygen, incorporation of two atoms of oxygen | 0.006092023 |
| C_D048-B12_90 | polyadenylate-binding protein 2 | Poly(A) RNA binding; protein binding; nucleotide binding | 0.004918883 |
| C_D048-C10_75 | ccaat enhancer binding protein (c ebp) gamma | Oxidoreductase activity; protein heterodimerization activity; sequence-specific DNA binding; RNA polymerase II distal enhancer sequence-specific DNA binding transcription factor activity; protein homodimerization activity; glucocorticoid receptor binding; transcription factor binding | 0.0000829 |
| C_D050-D9_68 | phosphogluconate dehydrogenase | Protein binding; NAD binding; phosphogluconate 2-dehydrogenase activity; NADP binding; carboxylic acid binding; phosphoglycerate dehydrogenase activity; phosphogluconate dehydrogenase (decarboxylating) activity | 0.00393318 |
| C_D050-G6_47 | tyrosyl-tRNA synthetase | Signal transducer activity; ATP binding; interleukin-8 receptor binding; tRNA binding; tyrosine-tRNA ligase activity | 0.00341505 |
| C_D050-G9_71 | arp2 actin-related protein 2 homolog | Actin binding; ATP binding | 0.000308979 |
| C_D051-D5_36 | actin-related protein 2 3 complex subunit 3 | Actin binding; structural constituent of cytoskeleton | 0.002281105 |
| C_D052-B10_74 | lysosomal alpha-mannosidase | Hydrolase activity, hydrolyzing O-glycosyl compounds; cation binding | 0.008117764 |
| C_D052-D2_12 | serine threonine-protein phosphatase 2a 56 kDA regulatory subunit epsilon isoform | Binding; protein phosphatase type 2A regulator activity | 0.0000377 |
| C_D052-E7_53 | retinol dehydrogenase 8-like | Oxidoreductase activity | 0.001820103 |
| C_D052-G6_47 | tyrosyl-tRNA synthetase | Signal transducer activity; ATP binding; interleukin-8 receptor binding; tRNA binding; tyrosine-tRNA ligase activity | 0.0000131 |
| C_G028-C04.b1.ab1 | mannose-binding lectin | Binding | 0.001966404 |
| C_G028-F07.b1.ab1 | glyceraldehyde-3-phosphate dehydrogenase | NAD binding; protein binding; peptidyl-cysteine S-nitrosylase activity; glyceraldehyde-3-phosphate dehydrogenase (NAD+) (phosphorylating) activity | 0.005958522 |
| C_G030-A10.b1.ab1 | ribosomal protein S28 | RNA binding; structural constituent of ribosome; protein binding; hydrolase activity | 0.004092152 |
| C_G030-B04.b1.ab1 | protein creg1-like | Transcription corepressor activity | 0.00453985 |
| C_G032-C03.b1.ab1 | alpha 1a | Protein domain specific binding; GTPase activity; protein heterodimerization activity; structural molecule activity; GTP binding | 0.009332587 |
| C_GS01JG02.b1.ab1 | calmodulin | N-terminal myristoylation domain binding; myosin VI head/neck binding; calmodulin-dependent protein kinase activity; protein domain specific binding; ATP binding; titin binding; thioesterase binding; calmodulin binding; calcium ion binding | 0.000248303 |
| C_GS01LB08.b1.ab1 | ribosomal protein S10 | - | 0.006656852 |
| C_GS01LC01.b1.ab1 | ribosomal protein l35a | Structural constituent of ribosome | 0.004901157 |
| C_GS01LF09.b1.ab1 | ribosomal protein l13a | Structural constituent of ribosome | 0.000964199 |
| C_GS01MG03.b1.ab1 | nonmuscle myosin essential light chain | Calcium ion binding | 0.001176085 |
| C_GS01OD11.b1.ab1 | peptidyl-prolyl cis-trans isomerase b-like | Peptide binding; unfolded protein binding; peptidyl-prolyl cis-trans isomerase activity | 0.001784229 |
| C_GS01QC09.b1.ab1 | ribosomal protein l12 | Structural constituent of ribosome | 0.000973752 |
| C_GS01RD06.b1.ab1 | protein | Protein kinase activity; ATP binding; transmembrane signaling receptor activity | 0.0000205 |
| C_GS01UF06.b1.ab1 | elongation factor 1-alpha | Translation elongation factor activity; GTPase activity; GTP binding | 0.000545348 |
| C_GS01UF11.b1.ab1 | selenoprotein w | NADH dehydrogenase activity | 0.005153163 |
| C_GS01WA02.b1.ab1 | tubulin alpha-3 | Protein binding; GTPase activity; structural molecule activity; GTP binding | 0.001371726 |
| C_GS01YA02.b1.ab1 | prostamide prostaglandin f synthase | Oxidoreductase activity, acting on the CH-OH group of donors, NAD or NADP as acceptor | 0.006540748 |
| C_GS01ZF02.b1.ab1 | catalase | Heme binding; catalase activity | 0.0000515 |
| C_GS01ZH08.b1.ab1 | catalase | Heme binding; catalase activity | 0.008117764 |
| C_MGE-A001-A12-post88- | ribosomal protein S7 | RNA binding; structural constituent of ribosome; protein binding | 0.000308979 |
| C_MGE-A003-C5-postH134 | translationally controlled tumor protein | Guanyl-nucleotide exchange factor activity | 0.006625622 |
| C_MGE-A004-C6-pstH9742 | yippee-like 2 | - | 0.003685499 |
| C_MGE-A005-A10-19372-T | bsd domain containing 1 | Protein binding | 0.008869549 |
| C_MGE-A005-G11-19386-T | protein | Dopamine beta-monooxygenase activity | 0.000802851 |
| C_mge-A008-H1-4817-T3 | protein | Lyase activity | 0.000503522 |
| C_MGE-A009-A7-57748-T3 | epidermal retinol dehydrogenase 2-like | Oxidoreductase activity; binding | 0.0000131 |
| C_MGE-A009-D7-57751-T3 | ricin b lectin | Binding | 0.008510252 |
| C_MGE-A010-E11-67384-T | h+ lysosomal v0 subunit c | Protein binding; hydrogen-exporting ATPase activity, phosphorylative mechanism; hydrogen ion transporting ATP synthase activity, rotational mechanism | 0.004023595 |
| C_MGE-A012-C12-86590-T | yorkie homolog | Transcription corepressor activity; protein binding; transcription coactivator activity | 0.005775417 |
| C_MGE-A014-D5-POST35-T | ribosomal protein S20 | Structural constituent of ribosome; RNA binding | 0.001340569 |
| C_MGE-A015-G5-post38-T | green fluorescent gfp-like protein | - | 0.002885039 |
| C_MGE-A016-C1-post2-T3 | atp synthase subunit mitochondrial | Hydrogen ion transmembrane transporter activity | 0.001009361 |
| C_MGE-A016-E8-post60-T | pituitary tumor-transforming 1 interacting protein | - | 0.0000515 |
| C_MGE-A018-C8-post58-T | activating transcription factor 4 | Transcription corepressor activity; protein C-terminus binding; DNA binding | 0.00578501 |
| C_MGE-A020-B11-post81- | 40S ribosomal protein s17 | Structural constituent of ribosome; hydrolase activity | 0.000816655 |
| C_MGE-A020-E7-post52-T | ribosomal protein s16 | Structural constituent of ribosome; RNA binding | 0.006857777 |
| C_MGE-A022-D12-post91- | eukaryotic translation initiation factor 5a | Translation elongation factor activity; protein N-terminus binding; translation initiation factor activity; ribosome binding; U6 snRNA binding | 0.003033695 |
| C_MGE-A022-E1-post4-T3 | calreticulin | Integrin binding; calcium ion binding; ubiquitin protein ligase binding; hormone binding; iron ion binding; unfolded protein binding; peptide binding; androgen receptor binding; mRNA binding | 0.000909474 |
| C_MGE-A022-F7-post53-T | sprouty homolog 3 | Protein binding | 0.0000131 |
| C_MGE-A027-F8-post61-T | legumain | Cysteine-type peptidase activity; protein serine/threonine kinase activity | 0.005300426 |
| C_mge-A030-G1-post6-T3 | wd repeat domain 1 | - | 0.00138196 |
| C_MGE-A034-A5-post32-T | activating transcription factor 3-like | DNA binding | 0.0000377 |
| C_mge-A036-C3-post18-T | bcl2-like 2 | Binding | 0.001886951 |
| C_mge-A037-A11-post80- | ras-like GTP-binding protein ypt1 | - | 0.000126144 |
| C_mge-A037-A7-post48-T | fev (ets oncogene family) | Sequence-specific DNA binding; sequence-specific DNA binding transcription factor activity | 0.001778282 |
| C_mge-A037-B6-post41-T | ubiquitin isoform cra_a | Protein binding | 0.007551734 |
| C_mge-A038-B7-post49-T | mapk mak mrk overlapping kinase | Cyclin-dependent protein kinase activity; advanced glycation end-product receptor activity; ATP binding; transmembrane signaling receptor activity | 0.0000131 |
| C_mge-A039-C12-post90- | aldo-keto reductase | Oxidoreductase activity | 0.00413252 |
| C_mge-A041-E10-post76- | lysosomal-associated transmembrane protein 4a | - | 0.001459291 |
| C_mge-A041-G4-post30-T | cox15 cytochrome c oxidase assembly protein | Cytochrome-c oxidase activity | 0.009745892 |
| C_mge-A042-G6-post46-T | sodium potassium adenosine triphosphatase | Metal ion binding; sodium:potassium-exchanging ATPase activity; ATP binding | 0.000617669 |
| C_mge-A044-G2-post14-T | cathepsin b | Cysteine-type endopeptidase activity | 0.00339857 |
| C_mge-A044-H11-post87- | calpain-9 isoform 1 | Cysteine-type endopeptidase activity | 0.005823429 |
| C_mge-A045-C3-post18-T | collagen alpha-1 chain | Protein binding | 0.002837244 |
| C_mge-A046-A5-post32-T | glutamate-cysteine catalytic subunit | ADP binding; glutamate binding; coenzyme binding; magnesium ion binding; RNA binding; glutamate-cysteine ligase activity | 0.002885039 |
| C_mge-A046-G10-post78- | musashi homolog 1 | Poly(U) RNA binding; nucleotide binding | 0.005677497 |
| C_mge-A047-A1-post0-T3 | brd4 protein | Protein binding; DNA binding | 0.002885039 |
| C_MGE-A049-A11-post80- | na+ k+ beta 3 polypeptide | - | 0.004426801 |
| C_MGE-A049-E7-post52-T | sgnh hydrolase | Cation binding | 0.00042723 |
| C_MGE-A049-H4-post31-T | cytoplasmic actin | ATP binding | 0.001986738 |
| C_MGE-A050-E2-post12-T | tho complex subunit 5 homolog | Protein binding | 0.001325587 |
| C_MGE-B011-G7-prawn54- | solute carrier family 25 (mitochondrial carrier phosphate carrier) member 3 | Binding; phosphate:hydrogen symporter activity; phosphate ion carrier activity | 0.001300382 |
| C_MGE-B012-G3-prawn22_ | serine threonine protein phosphatase in | Metal ion binding; phosphoprotein phosphatase activity | 0.000186408 |
| C_MGE-B014-H11-prawn87 | small nuclear ribonucleoprotein polypeptide g | RNA binding; protein binding | 0 |
| C_MGE-B015-H1-prawn7_T | death-associated protein 1 | - | 0.002113336 |
| C_mge-B016-B6-prawn41_ | abhydrolase domain containing 5 | Protein binding; triglyceride lipase activity | 0.0000205 |
| C_mge-B016-H12-prawn95 | alpha amylase | Glucosidase activity | 0.009281575 |
| C_mge-B018-E8-prawn60_ | interleukin enhancer binding factor 2 | Transcription regulator activity; DNA binding; double-stranded RNA binding; ATP binding; transferase activity; protein binding | 0.0000205 |
| C_mge-B019-F10-prawn77 | hla-b associated transcript 1 | ATP-dependent protein binding; ATP-dependent RNA helicase activity; identical protein binding; ATP binding; U4 snRNA binding; U6 snRNA binding | 0.0000377 |
| C_mge-B020-B10-prawn73 | cathepsin z | Cysteine-type endopeptidase activity | 0.0000377 |
| C_mge-B022-C5-prawn34_ | serine threonine-protein phosphatase 4 regulatory subunit 1-like | Protein binding | 0.005747289 |
| C_mge-B023-E1-prawn4_T | protein | Catalytic activity | 0.006396217 |
| C_MGE-B025-G9-prawn70_ | wee1-like protein kinase | Protein serine/threonine kinase activity; ATP binding | 0.0000131 |
| C_mge-B026-C2-prawn10_ | glycine n-methyltransferase | Glycine N-methyltransferase activity; folic acid binding | 0.005495339 |
| C_mge-B026-E7-prawn52_ | ATP synthase subunit mitochondrial | Hydrogen-exporting ATPase activity, phosphorylative mechanism; hydrogen ion transporting ATP synthase activity, rotational mechanism; ATP binding; proton-transporting ATPase activity, rotational mechanism | 0.001707682 |
| C_mge-B027-B4-prawn25_ | guanine nucleotide-binding protein gi gs gt subunit beta-1 | Protein binding; signal transducer activity | 0.0000131 |
| C_mge-B027-F4-prawn29_ | ribosomal protein l34 | Structural constituent of ribosome | 0.001429441 |
| C_mge-B031-B2-prawn9_T | cdc14 cell division cycle 14 homolog a ( cerevisiae) | Protein tyrosine phosphatase activity; protein tyrosine/serine/threonine phosphatase activity; protein binding | 0.000695955 |
| C_mge-B031-F3-prawn21_ | cytochrome b-c1 complex subunit 8 | - | 0.002001258 |
| C_mge-B031-H10-prawn79 | deoxyhypusine synthase | Protein binding; deoxyhypusine synthase activity | 0 |
| C_mge-B033-A8-prawn56_ | alpha-l- plasma | Alpha-L-fucosidase activity; cation binding | 0.000057 |
| C_mge-B033-G5-prawn38_ | clathrin light polypeptide | Peptide binding | 0.001387775 |
| C_mge-B034-D11-prawn83 | protein | DNA binding; protein binding | 0.000249887 |
| C_mge-B035-A8-prawn56_ | voltage-dependent anion channel 2 | Voltage-gated anion channel activity | 0.003299499 |
| C_mge-B035-B1-prawn1_T | family c2 unassigned peptidase (c02 family) | Cysteine-type peptidase activity; binding | 0.0000131 |
| C_mge-B035-C5-prawn34_ | 14-3-3 protein | Transcription corepressor activity; histone deacetylase binding; protein C-terminus binding; phosphoserine binding; protein domain specific binding; protein complex binding | 0.006563965 |
| C_mge-B035-D6-prawn43_ | nedd4 family-interacting protein 1 | Signal transducer activity; protein binding | 0.000215573 |
| C_mge-B036-E5-prawn36_ | riboflavin-binding protein | Riboflavin transporter activity | 0.00319536 |
| C_mge-B037-C2-prawn10_ | asparaginyl-trna cytoplasmic | Nucleic acid binding; asparagine-tRNA ligase activity; ATP binding; aspartate-tRNA ligase activity | 0.000959535 |
| C_mge-B037-D1-prawn3_T | hnrnp protein | mRNA binding; protein binding; nucleotide binding | 0.0000131 |
| C_MGE-B039-C4-prawn26_ | nadh dehydrogenase | Oxidoreductase activity, acting on NADH or NADPH | 0.001872979 |
| C_mge-B042-B4-prawn25_ | small nuclear ribonucleoprotein-associated protein n | Zinc ion binding; nucleosome binding; SUMO polymer binding; ubiquitin-protein ligase activity; transcription coactivator activity; identical protein binding; DNA binding; RNA binding; sequence-specific DNA binding transcription factor activity; transcription factor binding; androgen receptor binding | 0.001476194 |
| C_mge-B044-B2-prawn9_T | protein | DNA binding | 0.002648412 |
| C_mge-C001-C3-pre18_T3 | dolichyl-diphosphooligosaccharide-protein glycosyltransferase | Protein binding; dolichyl-diphosphooligosaccharide-protein glycotransferase activity | 0.002968669 |
| C_mge-C001-E6-pre44_T3 | cytochrome family subfamily polypeptide 5-like | Binding | 0 |
| C_mge-C001-G2-pre14_T3 | ferritin | Ferric iron binding; ferroxidase activity | 0.001269941 |
| C_mge-C002-B2-pre9_T3 | ribosomal protein l32 | Structural constituent of ribosome | 0.004505434 |
| C_mge-C002-H2-pre15_T3 | ribosomal protein l26 | Structural constituent of ribosome | 0.000599684 |
| C_mge-C003-B9-pre65_T3 | alpha-l- plasma | Alpha-L-fucosidase activity | 0.002341029 |
| C_mge-C003-D10-pre75_T | acidic leucine-rich nuclear phosphoprotein 32 family member a-like | Microtubule binding; phosphatase inhibitor activity | 0.002937586 |
| C_mge-C003-F11-pre85_T | adenosylhomocysteinase b | Adenosylhomocysteinase activity | 0.000256211 |
| C_mge-C004-C8-pre58_T3 | peroxiredoxin- mitochondrial-like | Peroxidase activity; cysteine-type endopeptidase inhibitor activity involved in apoptotic process | 0.000150061 |
| C_mge-C006-A7-pre48_T3 | red fluorescent protein | - | 0.00097422 |
| C_mge-C006-F10-pre77_T | protein disulfide isomerase | Oxidoreductase activity; isomerase activity | 0.000256211 |
| C_mge-C006-G7-pre54_T3 | placenta-specific gene 8 protein | - | 0.009983316 |
| C_mge-C007-A5-pre32_T3 | selenoprotein t | Selenium binding | 0.002447384 |
| C_mge-C010-B3-pre17_T3 | sodium-dependent phosphate transport protein 2b | Sodium-dependent phosphate transmembrane transporter activity | 0.008668163 |
| C_mge-C010-G3-pre22_T3 | cyclophilin-like protein | Hydrolase activity; peptidyl-prolyl cis-trans isomerase activity | 0.0000205 |
| C_mge-C011-F7-pre53_T3 | thymosin beta 4 | Actin monomer binding | 0.0000515 |
| C_mge-C012-D3-pre19_T3 | glutaredoxin domain-containing cysteine-rich protein cg12206-like | Electron carrier activity; protein disulfide oxidoreductase activity | 0.0000131 |
| C_mge-C012-H12-pre95_T | aldolase fructose-bisphosphate | Cytoskeletal protein binding; fructose binding; fructose-bisphosphate aldolase activity | 0.002748866 |
| C_mge-C013-D2-pre11_T3 | 40s ribosomal protein s9 | Structural constituent of ribosome; hydrolase activity; rRNA binding | 0.0000131 |
| C_mge-C014-D11-pre83_T | nucleoplasmin isoform 2 | - | 0.007387253 |
| C_mge-C014-H9-pre71_T3 | uv excision repair protein rad23 homolog b | Polyubiquitin binding; damaged DNA binding; single-stranded DNA binding | 0.001009361 |
| C_mge-C016-H8-pre63_T3 | capping protein (actin filament) muscle z- beta | Actin binding | 0.00010768 |
| S_D004-B9 | ubx domain-containing protein 7 | Protein binding | 0.004752097 |
| S_D006-B4 | sterol desaturase | Oxidoreductase activity; iron ion binding | 0.000121218 |
| S_D008-B11 | fad synthase | FMN adenylyltransferase activity | 0.0000377 |
| S_D008-D5 | udp-xylose and udp-n-acetylglucosamine transporter-like | UDP-xylose transmembrane transporter activity; UDP-N-acetylglucosamine transmembrane transporter activity | 0.000092 |
| S_D009-D3 | cyclic di-gmp-binding protein | - | 0.001608023 |
| S_D010-D1_4 | cysteine protease | Cysteine-type endopeptidase inhibitor activity; protein binding; cysteine-type endopeptidase activity | 0.000150061 |
| S_D013-H8_64 | tolloid-like 1 | - | 0.00647938 |
| S_D026-B1_2 | gtp-binding protein 8 | - | 0.000352635 |
| S_D027-B9_66 | tyrosine 3-monooxygenase tryptophan 5-monooxygenase activation iota polypeptide | Protein homodimerization activity; protein domain specific binding; protein heterodimerization activity | 0.002129728 |
| S_D027-D5_36 | ga11576- partial | N-acetyltransferase activity; protein binding; fatty acid binding | 0.00180466 |
| S_D039-A6_41 | cg10174 protein | - | 0.005272383 |
| S_D041-G8_63 | heat shock protein 70 | Chaperone binding; cysteine-type endopeptidase inhibitor activity involved in apoptotic process; unfolded protein binding; ubiquitin protein ligase binding; protein domain specific binding; ribosome binding; ATP binding; misfolded protein binding; ATPase activity; protein binding, bridging; calcium ion binding | 0.001340569 |
| S_D043-F5_38 | alpha-l- tissue | Cation binding; alpha-L-fucosidase activity | 0.0000515 |
| S_D052-F5_38 | a chain carbonic anhydrase ii complexed with 4- -n- -benzamide | Zinc ion binding | 0.00024579 |
| S_D053-C1_3 | tail fiber protein | - | 0.0000131 |
| S_GS01JF05.b1.ab1 | small ubiquitin-related modifier 1 precursor | Ubiquitin protein ligase binding | 0.001469311 |
| S_GS01LA12.b1.ab1 | ribosomal protein s15 | Nucleic acid binding; structural constituent of ribosome; protein binding | 0.00490042 |
| S_GS01PG05.b1.ab1 | signal sequence delta | - | 0.000633945 |
| S_MGE-A009-G12-57794-T3 | cryptochrome 1 (photolyase-like) | DNA photolyase activity; protein binding | 0.006775414 |
| S_MGE-A010-D9-67367-T3 | cdk5 regulatory subunit associated protein 3 | Protein binding | 0.0000205 |
| S_MGE-A010-H5-67339-T3 | alpha-aminoadipic semialdehyde dehydrogenase | L-aminoadipate-semialdehyde dehydrogenase activity; aldehyde dehydrogenase (NAD) activity; betaine-aldehyde dehydrogenase activity | 0.001573792 |
| S_MGE-A012-E9-86568-T3 | serine mitochondrial | Identical protein binding; glycine hydroxymethyltransferase activity; amino acid binding; L-allo-threonine aldolase activity; pyridoxal phosphate binding; methyltransferase activity | 0.003274507 |
| S_MGE-A018-G7-post54-T3 | predicted protein [Nematostella vectensis] | Zinc ion binding; nucleic acid binding | 0.008525026 |
| S_MGE-A021-F3-post21-T3 | 60S ribosomal protein l11 | Structural constituent of ribosome; protein binding; rRNA binding | 0.000249887 |
| S_MGE-A021-G4-post30-T3 | myocyte enhancer factor 2c protein | Histone deacetylase binding; activating transcription factor binding; protein kinase binding; protein heterodimerization activity; sequence-specific DNA binding; sequence-specific DNA binding transcription factor activity | 0.0000288 |
| S_MGE-A021-G9-post70-T3 | proteasome ( macropain) beta 1 | Protein binding; threonine-type endopeptidase activity | 0.000292526 |
| S_mge-A030-B2-post9-T3 | hypothetical protein A030-E11 [Acropora millepora] | Metal ion binding | 0.001429441 |
| S_MGE-A032-C8-post58-T3 | nfu1 iron-sulfur cluster scaffold mitochondrial-like | ATP-dependent helicase activity; nucleic acid binding; iron ion binding; ATP binding; 4 iron, 4 sulfur cluster binding | 0.000722689 |
| S_mge-A039-C8-post58-T3 | pdia4 protein | Electron carrier activity; isomerase activity; protein disulfide oxidoreductase activity | 0.0000131 |
| S_mge-A041-C7-post50-T3 | cubilin (intrinsic factor-cobalamin receptor) | Transporter activity; protein homodimerization activity | 0.004901157 |
| S_mge-A041-G6-post46-T3 | kinase ii delta | Calmodulin-dependent protein kinase activity; calcium-dependent protein serine/threonine phosphatase activity; calmodulin binding; ATP binding | 0.002534529 |
| S_mge-A046-E6-post44-T3 | zinc transporter 6 | Zinc ion transmembrane transporter activity | 0.0000131 |
| S_MGE-B014-A5-prawn32_T3 | mif4g domain-containing | - | 0 |
| S_MGE-B014-E5-prawn36_T3 | peptidyl-prolyl cis-trans isomerase cwc27 homolog | Peptidyl-prolyl cis-trans isomerase activity | 0.00849468 |
| S_mge-B018-C9-prawn66_T3 | n-acyl-phosphatidylethanolamine-hydrolyzing phospholipase d | Phospholipase activity | 0 |
| S_mge-C001-B7-pre49_T3 | hydrocephalus-inducing protein | - | 0 |
| S_mge-C002-D7-pre51_T3 | endoplasmic reticulum resident protein 44 | Protein binding; protein disulfide isomerase activity | 0.004449905 |
| S_mge-C003-B10-pre73_T3 | integrator complex subunit 4 | Protein binding | 0.004901157 |
| S_mge-C006-E2-pre12_T3 | lysosomal alpha-mannosidase-like | Alpha-mannosidase activity; carbohydrate binding; zinc ion binding | 0.0000131 |
| S_mge-C012-C5-pre34_T3 | vacuolar protein sorting-associated protein 53 homolog | - | 0.000186408 |
| S_mge-C015-E1-pre4_T3 | 17-beta-hydroxysteroid dehydrogenase 14 | Binding; oxidoreductase activity; structural constituent of ribosome | 0.005628797 |
| S_mge-C016-A9-pre64_T3 | protein | Oxidoreductase activity, acting on paired donors, with incorporation or reduction of molecular oxygen; metal ion binding; monooxygenase activity | 0.000155673 |
| S_mge-C016-C1-pre2_T3 | 1-acylglycerol-3-phosphate o-acyltransferase abhd5-like | Lysophosphatidic acid acyltransferase activity; triglyceride lipase activity | 0.000677966 |
