# Supplementary material for: High natural gene expression variation in the reef-building coral Acropora millepora: potential for acclimative and adaptive plasticity
Source: BMC Genomics. 2013 Apr 8;14:228. doi: 10.1186/1471-2164-14-228 (PMC3630057; doi:10.1186/1471-2164-14-228)
Supplement: Additional file 4 — Design of the microarray experiments. Dye swap was performed between colonies of the two intron 4-500 genotypes (shaded). Loop design was performed three times with different nubbins of each colony within each genotype. A total of 22 microarrays were used, as a replicate of one of the colonies of genotype 2 did not yield enough RNA. Colonies 1, 2, and 3 were genotyped as genotype 1 with intron 4-500. Colonies 4, 5, and 6 were genotyped as genotype 2 with intron 4-500. [file 1471-2164-14-228-S4.docx]

**Additional file 4** –**Design of the microarray experiments.**

Dye swap was performed between colonies of the two intron 4-500 genotypes (shaded). Loop design was performed three times with different nubbins of each colony within each genotype. A total of 22 microarrays were used, as a replicate of one of the colonies of genotype 2 did not yield enough RNA. Colonies 1, 2, and 3 were genotyped as genotype 1 with intron 4-500. Colonies 4, 5, and 6 were genotyped as genotype 2 with intron 4-500.

| **Colony** | **Nubbin** | **Label** | **Slide** |
| --- | --- | --- | --- |
| 1 | 1 | Cy5 | 007267 |
| 5 | 1 | Cy3 | 007267 |
| 5 | 1 | Cy5 | 007249 |
| 1 | 1 | Cy3 | 007249 |
| 2 | 1 | Cy5 | 007245 |
| 4 | 1 | Cy3 | 007245 |
| 4 | 1 | Cy5 | 007246 |
| 2 | 1 | Cy3 | 007246 |
| 3 | 1 | Cy5 | 007247 |
| 6 | 1 | Cy3 | 007247 |
| 6 | 1 | Cy5 | 007248 |
| 3 | 1 | Cy3 | 007248 |
| 1 | 3 | Cy5 | 007271 |
| 2 | 4 | Cy3 | 007271 |
| 2 | 3 | Cy5 | 007266 |
| 3 | 2 | Cy3 | 007266 |
| 3 | 1 | Cy5 | 007268 |
| 1 | 1 | Cy3 | 007268 |
| 1 | 1 | Cy5 | 007284 |
| 2 | 2 | Cy3 | 007284 |
| 2 | 4 | Cy5 | 007270 |
| 3 | 4 | Cy3 | 007270 |
| 3 | 2 | Cy5 | 007281 |
| 1 | 2 | Cy3 | 007281 |
| 1 | 2 | Cy5 | 007286 |
| 2 | 3 | Cy3 | 007286 |
| 2 | 2 | Cy5 | 007285 |
| 3 | 1 | Cy3 | 007285 |
| 3 | 4 | Cy5 | 007279 |
| 1 | 3 | Cy3 | 007279 |
| 4 | 1 | Cy5 | 007272 |
| 6 | 1 | Cy3 | 007272 |
| 6 | 4 | Cy5 | 007283 |
| 5 | 4 | Cy3 | 007283 |
| 5 | 2 | Cy5 | 007276 |
| 4 | 2 | Cy3 | 007276 |
| 4 | 2 | Cy5 | 007277 |
| 6 | 2 | Cy3 | 007277 |
| 6 | 2 | Cy5 | 007280 |
| 5 | 2 | Cy3 | 007280 |
| 5 | 4 | Cy5 | 007278 |
| 4 | 3 | Cy3 | 007278 |
| 4 | 3 | Cy5 | 007282 |
| 6 | 4 | Cy3 | 007282 |
